# Detection and Molecular Characterization of Canine Babesiosis Causative Agent *Babesia canis* in Naturally Infected Dogs in the Dobrogea Area (Southeastern Romania)

**DOI:** 10.3390/life13061354

**Published:** 2023-06-09

**Authors:** Mariana Ionita, Laurentiu Leica, Marion Wassermann, Emanuel Mitrea, Isabela Madalina Nicorescu, Ioan Liviu Mitrea

**Affiliations:** 1 Department of Parasitology and Parasitic Diseases & Animal Biology, Faculty of Veterinary Medicine, University of Agronomic Sciences and Veterinary Medicine of Bucharest, 11464 Bucharest, Romania; ionitamary@yahoo.com (M.I.); leica.laurentiu@yahoo.com (L.L.); mitread.emanuel@gmail.com (E.M.); isabela_nicorescu@yahoo.com (I.M.N.); 2 Parasitology Unit, Institute of Biology, University of Hohenheim, 70599 Stuttgart, Germany; marion.wassermann@uni-hohenheim.de

**Keywords:** canine babesiosis, *Babesia canis*, *Babesia vogeli*, molecular detection, sequence analysis, southeastern Romania

## Abstract

Canine babesiosis is an emerging tick-borne disease of major veterinary concern in Europe. Its prevalence has increased in the last two decades and is spreading rapidly toward the north. The aim of this study was to investigate the genetic diversity of *Babesia* spp. strains isolated from naturally infected dogs in a tick-endemic area (Dobrogea) in southeastern Romania. For this purpose, a total of twenty-three samples from dogs diagnosed with various clinical forms of babesiosis, evaluated by means of clinical history, physical examination, and hematological tests, were subjected to a molecular investigation using PCR, sequencing analysis, and genetic characterization. A microscopic examination of thin Diff-quick-stained blood smears revealed large intra-erythrocytic *Babesia* piroplasms in all dogs. The PCR and sequencing analysis results indicated the presence of *Babesia canis* in 22 dogs (95.7%) and *Babesia vogeli* in 1 dog (4.3%). Among the *B. canis* isolates, two genotypes were distinguished based on two nucleotide substitutions (GA→AG) observed in the 18S rRNA gene sequences (at positions 609 and 610), with the AG genotype predominating (54.5% of samples), while the GA variant was identified in 9.1% of samples. In the remaining isolates (36.4%), both variants were identified. The *B. vogeli*-positive dog also tested positive for antibodies against *Ehrlichia canis* and displayed severe disease. This study reports, for the first time, the presence of genetically heterogenic *B. canis* strains in dogs with clinical babesiosis in Romania. These findings provide a basis for future studies on the relationship between the genetic structure of the causative agents of canine babesiosis in Romania and the course of the disease.

## 1. Introduction

Canine babesiosis (CB) is a tick-borne disease that significantly impacts dogs’ health worldwide [1,2]. It is caused by large and small intra-erythrocytic protozoa (Apicomplexa: Piroplasmida) vectored by different tick species. The prevalence of babesiosis and its geographical distribution generally vary according to the tick spectrum in a given area [3,4].

Currently, CB is increasingly reported in many European countries as an emerging and rapidly expanding disease [2,5]. Moreover, several recent studies have reported the spreading and/or occurrence of CB in northern countries, such as Belgium [6], the Netherlands [7], and Norway [8], which are related to the geographic expansion of the tick vector *Dermacentor reticulatus* [9]. These findings emphasize the need for the continuous monitoring of CB in both endemic and non-endemic areas.

The clinical presentation of babesiosis in dogs may range from subclinical to acute, even fatal, disease [3]. The severity of infection is mainly attributed to the pathogenicity of the causative *Babesia* species [10,11]; however, other factors related to the dog’s age, immunity, and concomitant infections also play a role in the variable pathogenicity of piroplasms infecting dogs [12,13].

In addition to the *Babesia*-induced hemolytic anemia and hypoxia, some dogs develop a complicated babesiosis characterized by immune-mediated hemolytic anemia (IMHA) and/or signs of inflammatory reactions [14,15]. Subsequently, abnormalities seen in complicated canine babesiosis cases include hepatopathy, acute kidney injury (AKI), acute respiratory distress syndrome (ARDS), cerebral babesiosis, pancreatitis, rhabdomyolysis, and myocardial dysfunction [16].

Traditionally, laboratory diagnosis has relied on the microscopic detection of intra-erythrocytic piroplasms. However, this technique is laborious, time-consuming, and has limitations, depending on the disease stage. For instance, this method is more accurate during the acute stage of the disease [1]. On the other hand, the molecular diagnosis shows high sensitivity and specificity, making it useful and adequate to detect low parasitemia levels (e.g., during the subclinical or chronic disease stages) [17]. The amplification of parasite DNA indicates evidence of infection; moreover, the sequencing results are more reliable and allow differentiation between the large *Babesia* piroplasm species (i.e., *B. canis* and *B. vogeli*), which are morphologically indistinguishable [10,18,19].

In Romania, the epidemiology of CB has shown a continuous dynamic in the last two decades, progressing from sporadic cases to endemic areas, such as southeastern Romania, where an increasing number of clinical CB cases are reported [20,21,22,23]. However, limited data are available on the genetic characterization of the *Babesia* species associated with various clinical presentations of CB in Romania. Therefore, the aim of the present study was to provide new insights into the molecular epidemiology of clinical canine babesiosis, with contributions from the molecular detection, sequencing analysis, and genetic characterization of *Babesia* strains isolated from naturally infected Romanian dogs.

## 2. Materials and Methods

### 2.1. Study Design

Twenty-three client-owned dogs with clinical babesiosis were included in this study. These dogs were exclusive patients of the study clinic, originated from the Dobrogea region (southeastern Romania), and had no history of travelling outside Romania.

Dobrogea is a historical region in the coastal area of the Black Sea in southeastern Romania, located between the lower Danube River and the Black Sea.

Data about each dog’s breed, age, lifestyle, tick infestation history, and outdoor access were registered.

The clinical history, physical examination, and laboratory testing were corroborated to confirm the diagnosis.

A molecular analysis of the partial 18S rRNA gene was performed for species identification and genetic characterization.

### 2.2. Samples and Laboratory Investigations

Whole peripheral (cephalic vein) blood samples were collected from the dogs and subjected to laboratory investigations.

The diagnosis of babesiosis was established by the presence of intra-erythrocytic parasites in thin blood smears stained with Diff-quick stain [20].

To assess the hemato-biochemical alterations, selected hematological (blood cell count) and biochemical parameters were determined using automatic hematology (Abacus Vet Jr., Diatron MI ZRT, Budapest, Hungary) and biochemistry (SPOTCHEM^™^ EZ SP-4430, Arkray, Europe, Amstelveen, The Netherlands) analyzers, respectively.

Depending on the clinical presentation (clinical signs such as fever, depression, anorexia, and pale mucous membranes) and the severity of anemia, the cases were classified as mild or severe disease. The severity of anemia (lower values for the packed cell volume (PCV) and/or hemoglobin (Hgb)) was defined as follows: mild (30% ≤ PCV < 37% and/or 10 ≤ Hgb < 12 g/dL), moderate (20% ≤ PCV < 30% and/or 5 ≤ Hgb < 10 g/dL), or severe (PCV < 20% and/or Hgb < 5 g/dL).

The severity of thrombocytopenia (lower values for the platelet count (PLT)) was defined as follows: mild (100 ≤ PLT < 200 × 10^9^/L), moderate (50 ≤ PLT < 100 × 10^9^/L), or severe (PLT < 50 × 10^9^/L).

Additionally, we classified the cases based on detected clinicopathological changes as (i) uncomplicated babesiosis when varying degrees of hemolytic anemia were present, (ii) complicated babesiosis with single organ dysfunction, and (iii) complicated babesiosis with multiple organ dysfunction syndrome (MODS), as previously described [14,23,24,25]. Briefly, for defining complicated babesiosis, in addition to the hemolytic anemia, the following criteria for organ dysfunction were used: (i) for acute renal injury: creatinine ≥ 1.5 mg/dL; (ii) for hepatopathy: two elevated liver enzymes (alkaline phosphatase (AP) > 280 U/L and alanine aminotransferase (ALT) > 60) and/or aspartate aminotransferase (AST) or a single elevated enzyme (AP > 560 U/L or ALT > 120 U/L); (iii) clinical signs of acute respiratory distress syndrome (ARDS). MODS was considered when at least two of the above dysfunctions were present.

To investigate potential coinfections and the impact of other vector-borne diseases on clinical presentation severity, the dogs were also tested using a commercial ELISA-based kit (SNAP^®^ 4Dx^®^, IDEXX Laboratories, Inc. Westbrook, ME, USA). This kit can simultaneously detect circulating antibodies against *Anaplasma* spp., *Borrelia burgdorferi* sensu lato, and *Ehrlichia canis* (three tick-borne pathogens) and the circulating antigen of *Dirofilaria immitis* (a mosquito-borne pathogen).

EDTA-anticoagulated peripheral blood was stored at −20 °C for subsequent DNA extraction and analysis.

### 2.3. DNA Isolation, PCR Amplification, and Sequencing

Twenty-three dog blood samples were subjected to the molecular detection of *Babesia* spp. DNA and a sequence analysis. For this, total genomic DNA was extracted from 200 μL of EDTA whole blood from each sample using a DNeasy Tissue kit (Qiagen AG, Basel, Switzerland) according to the manufacturer’s instructions. The DNA samples were stored at −20 °C until processing.

For the molecular detection of *Babesia* DNA, a conventional polymerase chain reaction (PCR) method was conducted using a genus-specific set of primers that amplify a specific region of the 18S rRNA gene of *Babesia* spp. of approx. 450 bp [26].

The PCR reactions were performed on a Mini Thermal Cycler MJ (Bio-Rad^®^, Hercules, CA, USA) in a reaction volume of 50 μL containing 25 μL of 2X Taq PCR Master Mix (Qiagen AG, Basel, Switzerland), 0.5 μM of each primer, and 5 μL of the extracted DNA as a template. The cycle conditions were described previously [20].

The PCR amplicons were visualized on a 2% agarose gel and stained with ethidium bromide.

Positive PCR products were purified using a QIAQuick PCR purification kit (Qiagen, Hilden, Germany) and subsequently sequenced in both directions (CeMIA, Larissa, Greece).

### 2.4. DNA Sequence and Phylogenetic Analysis

The obtained electropherograms were viewed and edited using GENtle (Manske, University of Cologne). The sequences were compared to the deposited reference sequences in GenBank using the online tool BLAST (National Center for Biotechnology Information; https://www.ncbi.nlm.nih.gov/ accessed on 6 May 2023).

Phylogenetic analyses were performed with reference sequences of *Babesia canis* from GenBank using the neighbor-joining method with the Jukes–Cantor substitution model. *Babesia gibsoni* was used as an outgroup. The phylogenetic tree was tested with 1000 bootstrap replications. The analysis was conducted in MEGA X [27].

### 2.5. Statistical Analysis

For the statistical analysis, Fisher’s exact test was used (Quantitative Parasitology 3.0 free software) [28]. *p* ≤ 0.05 was considered statistically significant.

## 3. Results

### 3.1. Clinical Cases

A total of 23 dogs (17 males and 6 females) of different breeds, varying from 3 months to 13 years of age (mean age = 4.2 years; median = 2.0), that displayed various clinical signs of babesiosis were evaluated by means of clinical history, physical examination, and hematological testing.

Blood samples obtained from the dogs were also subjected to PCR, a sequencing analysis, and genetic characterization. All 23 dogs were diagnosed between February and June 2019, predominantly in April, May, and June (n = 17) but also in February and March (3 dogs each). A summary of the epidemiological data and the clinical presentation is presented in Table 1.

A microscopic examination of thin stained blood smears from all 23 samples revealed single and/or paired intra-erythrocytic microorganisms that were morphologically consistent with large *Babesia* piroplasms.

Two dogs also tested serologically positive for antibodies against other tick-borne pathogens, namely, *E. canis* and *Anaplasma* spp.

### 3.2. PCR and DNA Sequence Analysis

Positive PCR results were obtained for all canine blood samples. The length of the PCR product corresponded to the expected size, at approximately 450 bp.

All PCR products were sequenced, and the BLASTn analysis of the sequences revealed the presence of two *Babesia* species, namely, *Babesia canis* in 22/23 samples (95.7%) and *Babesia vogeli* in 1 sample (4.3%) (Table 1).

The nucleotide sequence analysis showed that two different genetic variants could be distinguished among the *B. canis* isolates based on two nucleotide substitutions (GA→AG) occurring in the 18S rRNA gene sequence. The two genotypes differ at two consecutive sites, 129 and 130, in our gene fragment [26], which, if the complete *B. canis* 18S rRNA gene sequence is taken as a reference (GenBank accession No. AY072926) [29], are located at nucleotide positions 609 and 610 of the whole gen (Table 2). Twelve samples (54.5%; 12/22) showed the bases adenine and guanine (AG), while two samples (9.1%; 2/22) showed guanine and adenine (GA) at these positions. In the remaining 8/22 samples (36.4%), both adenine and guanine were observed at each of these positions in the electropherogram, displaying A/G double peaks (RR), which may indicate infection with both variants.

The BLAST analysis of the AG sequences showed 100% similarity to isolates previously identified in Romania [20] as well as other countries, such as Slovenia [30], Hungary [31], Russia [32], Poland [33], Croatia [34], Estonia [35], Austria [36], the Republic of Moldavia [37], and Turkey (MN704759.1; KF499115.1).

**Figure 1:**
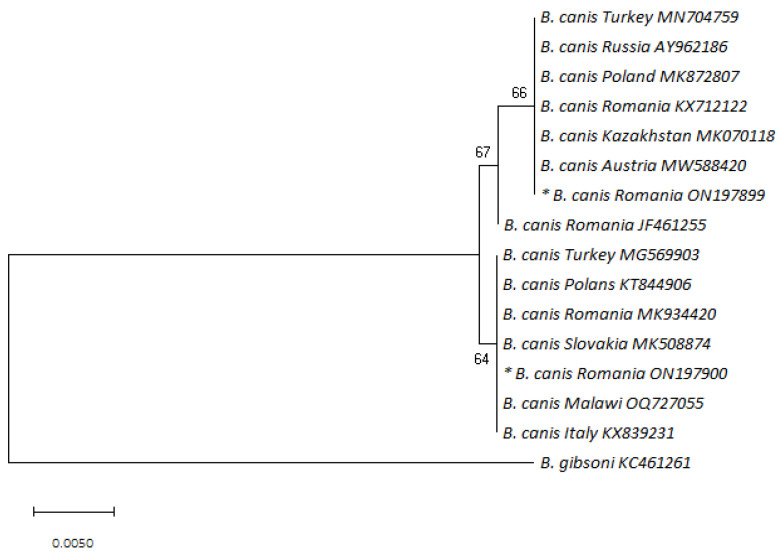
Phylogenetic tree of *Babesia canis* based on neighbor-joining analysis of 447 bp long 18S rRNA fragments; *B. gibsoni* was used as an outgroup. Sequence names consist of the species, the country of origin, and the corresponding accession number. Bootstrap values are shown on the nodes. The sequences discovered in the present study are marked with an asterisk (nucleotide sequence * ON197899 corresponds to the AG variant; nucleotide sequence * ON197900 corresponds to the GA variant).

The GA variant also showed identity with isolates from other countries, such as France [38], Italy [29], the Netherlands [7], Croatia [34], Lithuania [39], Poland [40], Slovakia (MK5088770), Spain (MK591947), Latvia [41], and Russia [32]. The sequence alignment of the partial 18S rDNA sequence of the *B. canis* isolates analyzed in this study that showed differences (AG/GA/RR) in comparison with the reference gene sequence (AY072926) is depicted in Table 2. The phylogenetic analysis showed that the sequences from this study were identical to other *B. canis* isolates that display the AG and GA genotypes (Figure 1).

The *B. vogeli* sequence was identical to the reference sequence JF461252, which was previously detected in Romania [20]. This genetic variant was also found in Egypt [42] and Israel [43].

The partial 18S rRNA sequences of the AG and GA *B. canis* variants and the *B. vogeli* variant were deposited in GenBank (accession numbers: ON197899.1, ON197900.1, and ON197901.1, respectively).

### 3.3. Clinicopathological Findings in B. canis-Infected Dogs

All *B. canis*-positive dogs were symptomatic, displaying clinical signs consistent with canine babesiosis. All the dogs showed lethargy and anorexia, while fever and hemoglobinuria were registered in 95.5% (21/22) and 68.2% (15/22) of the cases, respectively.

The dogs showed various degrees of clinical presentation: mild in 27.3% (6/22), moderate in 31.8% (7/22), and severe in 40.1% (9/22) of cases. Additionally, according to the pathological findings (hematological and biochemical abnormalities), 40.9% (9/22) of the dogs were diagnosed with uncomplicated babesiosis, whereas 59.1% (13/22) of the dogs were diagnosed with complicated babesiosis, with either single organ dysfunction (SOD) (40.9%; 9/22) or multiple organ dysfunction syndrome (MODS) (18.2%; 4/22) (Table 1). Based on the defining criteria, the following complications were diagnosed: acute renal injury was diagnosed in nine dogs as single organ dysfunction (n = 5) or MODS (n = 4); hepatopathy was diagnosed in eight dogs as single (n = 4) or multiple (n = 4) organ dysfunction; and ARDS was diagnosed in one case as MODs.

The most common hematological changes in *B. canis*-positive dogs included normocytic normochromic anemia (63.6%; 14/22) and thrombocytopenia (59.1%; 13/22). Dogs with anemia were classified as mild (14.2%), moderate (57.1%), and severe (28.6%). Of the dogs with thrombocytopenia, severe thrombocytopenia (PLT < 50 × 10^9^/L) was registered in >50% (53.8%; 7/13) of cases (Table 3).

Regarding the biochemical abnormalities, the most frequent changes included hyperbilirubinemia in 72.7% (16/22) of cases and azotemia (increased serum creatinine and/or blood urea nitrogen (BUN)) in 59.1% (13/22) of cases (for nine dogs, concurrent increased creatinine and BUN were registered, while four showed increased BUN). Hepatic enzymes (alanine aminotransferase (ALT), alkaline phosphatase (AP), and/or aspartate aminotransferase (AST)) were elevated in 45.4% (10/22) of cases, while pancreatic amylase was increased in 4.5% (2/22) of dogs.

The clinicopathological findings were compared to the *B. canis* genotypes, and a statistically significant relationship was found between the genotype and clinical presentation severity (*p* = 0.02), severe anemia, and severe thrombocytopenia (*p* < 0.05), with a higher severity for the GA genotype (Table 2). However, since the GA genotype was found in only two cases, further studies are necessary to demonstrate the genotype-associated risks for severe disease.

No statistically significant correlation between the genotype and complications (complicated babesiosis with SOD and/or MOD) was found (*p* > 0.05).

Table 3 summarizes the data on the most frequent complications and hematological and biochemical alterations among *B. canis*-positive dogs.

### 3.4. Clinicopathological Findings in the B. vogeli-Infected Dog

The *B. vogeli*-positive dog (a seven-year-old male mongrel) also tested positive for antibodies against *E. canis*, a tick-borne pathogen also vectored by *R. sanguineus*. This dog had severe anemia, thrombocytopenia, and elevated hepatic (ALT and AST) and pancreatic (AMY) enzyme values.

The *B. vogeli*-positive dog displayed a severe disease characterized by lethargy, anorexia, fever (39.6 °C), severe anemia (PCV = 9.1% and Hgb = 2.5 g/dL), and severe thrombocytopenia (PLT = 17 × 10^9^/L).

## 4. Discussion

As demonstrated in previous studies, CB is recognized as an endemic disease in southeastern Romania, including the study area [20,23]. The findings of the present study confirm the presence of both large *Babesia* piroplasm species, namely, *B. canis* and *B. vogeli*, in dogs with clinical babesiosis [44,45,46].

*B. canis* is recognized as the most common agent of canine babesiosis in Europe [2]. Our molecular analysis also identified *B. canis* as the most common causative agent of babesiosis in symptomatic dogs in the investigated region.

Additionally, to our knowledge, this is the first molecular study on the genetic diversity of *B. canis* strains isolated from naturally infected dogs in Romania by amplifying and sequencing a portion of the 18S ribosomal rRNA gene. Subsequently, our results indicated the presence of two different genotypes of *B. canis* in Romanian dogs. These genotypes differ by two nucleotide substitutions (GA→AG) at positions 129 and 130 of the partial 18S rRNA gene sequence obtained in our study, as shown in Table 2.

Previous studies have also observed that *B. canis* can differ at these sequence positions. Although they did not find differences in the entire length of the 18S rRNA gene between Croatian, Italian, and Polish isolates, their complete 18S rRNA sequence (AY072926) served as a reference for other studies [29]. Thus, when comparing their Slovenian samples to the reference AY072926, Duh et al. [30] found three genotypes differing at two consecutive positions, 609 and 610 (GA/AG/AA), of the complete gene [29,30]. Since then, other molecular surveys have reported on this single-nucleotide polymorphism as an intra-specific genetic variation of *B. canis* isolates from different European countries, such as Poland [33,47], Croatia [34], Switzerland [48], Lithuania [39,49], Latvia [41], and Russia [32].

Currently, four genotypes of the *B. canis* 18S rRNA gene with nucleotide substitutions (GA/AG/AA/TT) at these positions are reported in Europe. However, since most studies, including this one, have not examined the complete 18S rRNA gene, it cannot be excluded that there are additional genotypes. The *B. canis* genotypes found in our study correspond to the previously reported molecular findings on the nucleotide polymorphism related to the GA→AG substitutions (Table 2).

Infections with only the AG genotype were most common and were revealed in 54.5% (12/22) of the isolates in our study. The AG sequence was identical to dog isolates previously identified in Romania [20] and others reported in Central and Eastern Europe with prevalence values ranging from 23.4% (23/98 isolates) in Croatia [34] to 35.0% (84/240 isolates) in Poland [47] and up to 54.5% (6/11 isolates) in Slovenia [30]. The AG genotype was also reported as the most prevalent, at 80.9% (17/21 isolates), among dog isolates in Russia [32].

The GA variant, displaying guanine and adenine at the aforementioned positions, was found in our study as a single infection in only two samples (9.1%). This genetic variant was detected for the first time in Romanian dogs but is considered common in other European countries where it is found most often, such as Croatia, at 58.1% (57/98 isolates); Poland, at 44.6% (107/240 isolates); and Lithuania, at 34.2% (13/38 isolates) [34,39,47].

The double peaks (RR) found in eight of the dog isolates (36.4%) in the present study have also previously been described in studies from Croatia (18.3%; 18/98 isolates) [34], Lithuania (65.8%; 25/38 isolates) [39], Latvia (91%; 39/43 isolates) [41], and Switzerland (100%; 19/19 isolates) [48]. The presence of both nucleotides (A/G, RR) at these positions can be explained either by a mixed infection with two *B. canis* genotypes or by the possibility of genetic heterogeneity occurring among the 18S rRNA gene copies [34,48]. In this respect, investigations performed on some *Babesia* species identified three (or four) copies of the ssrRNA genes, i.e., three ribosomal RNA (rRNA) transcription units (rDNA units) in *Babesia bigemina* and *Babesia bovis* and four in *B. canis* [50,51]. However, very limited heterogeneity in the ssrRNA copies within each species was reported [50]. Since all dogs in our study originated from the same area and ticks could harbor different *B. canis* genotypes, mixed infections seem to be the more likely explanation of the present finding.

The clinicopathological changes in *B. canis*-infected dogs in our study were consistent with canine babesiosis. The most common abnormalities were anemia (63.6%), thrombocytopenia (59.1%), hyperbilirubinemia (72.7%), and azotemia (59.1%).

When the clinicopathological changes were compared with the *B. canis* genotypes, a statistically significant correlation was found between the genotype and the severity of the clinical presentation, anemia, and thrombocytopenia (*p* < 0.05). However, no correlation between the genotype and babesiosis complications (*p* > 0.05) was found. In a recent study on the genetic analysis of *B. canis* strains in dogs in Poland, there were four polymorphic groups reported based on two nucleotide substitutions in the 18S rRNA gene fragment (GA, AG, TT, and a fourth with variable nucleotides). Of these, the GA (107 isolates), AG (84 isolates), and TT (7 isolates) genotypes were associated with acute babesiosis. In contrast, representatives of the TT (31 isolates) and fourth (11 isolates) groups were associated with atypical and subclinical babesiosis, respectively [47]. Similar studies analyzing the clinicopathological findings in 26 symptomatic dogs with acute babesiosis did not determine any relationship between the *B. canis* genotypes GA/RR and the disease’s severity [41]. In our study, the GA genotype was found to be more severe in relation to anemia and thrombocytopenia than the AG or mixed genotypes. However, the low number of single GA genotypes identified (only two isolates) could have biased the results. Therefore, the relationship between the genetic structure of the protozoan pathogen and the disease course in dogs still requires future research studies, particularly regarding its diagnostic and/or prognostic value.

Apart from *B. canis*, we also molecularly detected *B. vogeli* in naturally infected dogs.

*B. vogeli* is vectored by *Rhipicephalus sanguineus* [10] and has mainly been detected in tropical and subtropical areas of Africa, Europe, Asia, and North and South America [42,52]. Although *B. vogeli* is considered the least pathogenic large species of *Babesia*, leading to mild or moderate and often clinically unapparent or subclinical infections, at least in adult dogs, it can cause severe disease in young dogs and when coinfections occur [53,54,55].

The clinicopathological findings of our study are in agreement with a recent study of the clinicopathological profile of *B. vogeli* infection and *B. vogeli*/*E. canis* coinfection [55]. In their study, a hematological data comparison between the only *B. vogeli*-positive group and the *E. canis* coinfection group showed statistically significant differences in red blood cell (RBC) parameters, including the RBC count, hemoglobin concentration, hematocrit, and RBC distribution width. However, the pathogenic mechanisms underlying this infection, such as the destruction of RBCs, require further investigation.

The mixed infection in the dog with *B. vogeli* and *E. canis* found in our study suggests a more severe clinical disease. Coinfections with other vector-borne pathogens have also been reported in recent studies of dogs in southeastern Romania [21,22]. Since coinfections often result in higher pathogenicity and complications, coinfected cases are of major clinical relevance [56].

These findings suggest that mixed infections, namely, *Babesia* spp. and other vector-borne pathogens, can be expected in Romanian dogs. Therefore, the simultaneous testing of dogs for multiple vector-borne pathogens is required for accurate diagnosis and prognosis and effective therapy.

The detection of both causative agents of canine babesiosis in Romanian dogs, namely, *B. canis* and *B. vogeli*, is in agreement with the sympatric occurrence and distribution of the two different tick vectors for the two *Babesia* species, i.e., *D. reticulatus* for *B. canis* and *R. sanguineus* for *B. vogeli*, in the study area, as recently reported [23,57]. *D. reticulatus* and *R. sanguineus* are the most common ticks infesting dogs in Romania, especially in southeastern Romania. The tick population has reportedly increased in recent years, including in the investigated area [23,57,58]. As none of the examined dogs had traveled outside Romania, we presumed that the *B. canis* and *B. vogeli* infections were autochthonously acquired.

## 5. Conclusions

This study reports, for the first time, the presence of two different *B. canis* genotypes in dogs with clinical babesiosis in Romania. The mixed infection with *B. vogeli* and *E. canis* of a symptomatic dog suggests that the simultaneous testing of dogs for multiple vector-borne pathogens is required for accurate diagnosis and prognosis and effective therapy. These findings provide a basis for future studies on the relationship between the genetic structure of the causative agents of canine babesiosis in Romania and the course of the disease.

## Figures and Tables

**Table 1:** Descriptive epidemiological data on microscopically (blood smear) and molecularly (PCR and sequencing) *Babesia*-positive dogs in southeastern Romania (data are stratified by clinicopathological groups).

Variable	No. (%)	Clinical Presentation			Uncomplicated and Complicated (C-) Babesiosis		
		Mild	Moderate	Severe	Uncomplicated	* C-SOD	** C-MOD
Total dogs (n = 23)		6 (26.1)	7 (30.4)	10 (43.5)	10 (43.5)	9 (39.1)	4 (17.4)
Age (years)							
<1	6 (26.1)	3 (50.0)	2 (33.3)	1 (16.7)	4 (66.7)	2 (33.3)	0
1–3	8 (34.8)	2 (25.0)	3 (37.5)	3 (37.5)	3 (37.5)	4 (50.0)	1 (12.5)
4–6	3 (8.7)	0	0	3 (100)	0	1 (33.3)	2 (66.7)
7–9	2 (8.7)	1 (50.0)	0	1 (50.0)	1 (50.0)	1 (50.0)	0
≥10	4 (17.4)	0	2 (50.0)	2 (50.0)	2 (50.0)	1 (25.0)	1 (25.0)
Sex							
male	17 (73.9)	5 (29.4)	2 (11.8)	10 (58.8)	8 (47.1)	5 (29.4)	4 (23.5)
female	6 (26.1)	1 (16.7)	5 (83.3)	0	2 (33.3)	4 (66.7)	0
Breed							
pure breed	17 (73.9)	4 (23.5)	6 (35.3)	7 (41.2)	7 (41.2)	6 (35.3)	4 (23.5)
mixed breed	6 (26.1)	2 (33.3)	1 (16.7)	3 (50.0)	3 (50.0)	3 (50.0)	0
*Babesia* species							
*Babesia vogeli*	1 (4.4)	0	0	1 (100)	1 (100)	0	0
*Babesia canis*	22 (95.6)	6 (27.3)	7 (31.8)	9 (40.9)	9 (40.9)	9 (40.9)	4 (18.2)
*B. canis* genotypes							
AG	12 (54.5)	4 (33.3)	7 (58.3)	1 (8.4)	6 (50)	6 (50)	0
GA	2 (9.1)	0	0	2 (100)	0	1 (50.0)	1 (50.0)
RR	8 (36.4)	2 (25.0)	0	6 (75.0%)	3 (37.5)	2 (25.0)	3 (37.5)
*p*-value		*1.000*	*0.012*	*0.002*	*0.571*	*0.571*	*0.051*

* C-SOD: complicated with single organ dysfunction; ** C-MOD: complicated with multiple organ dysfunctions.

**Table 2:** Differences in the nucleotide sequences of the 18S rRNA gene of the *Babesia canis* isolates analyzed in this study (#) and the partial *B. canis* 18S rRNA gene reference (AY072926). Positions are numbered with reference to the full-length sequence.

GenBank Accession No.	Nucleotide Position	
	609	610
AY072926.1	G	A
#ON197899.1	A	G
#ON197900.1	G	A
#Mixed RR	G/A	G/A

**Table 3:** Relative frequencies of common pathological changes in 22 *Babesia canis*-positive dogs in southeastern Romania.

	Pathological Changes in *Babesia canis*-Positive Dogs, Stratified by *B. canis* 18S rRNA Genotypes: Number of Dogs; Percentage (%)												
	Anemia				Thrombocytopenia				* T-bil↑	**Azotemia**	** H **↑**	*** P **↑**	**MODs**
	Total	Mild	Moderate	Severe	Total	Mild	Moderate	Severe					
Total (n = 22)	14 (63.6)	2 (14.2)	8 (57.1)	4 (28.6)	13 (59.1)	*3 (23.1)*	*3 (23.1)*	*7 (53.8)*	16 (72.7)	13 (59.1)	10 (45.4)	1 (4.5)	4 (18.2)
*B. canis* genotypes													
AG (n = 12)	5 (41.7)	1 (8.33)	4 (33.3)	0	4 (33.3)	2 (16.7)	1 (8.3)	1 (8.3)	8 (66.7)	5 (41.7)	5 (41.7)	0	0
GA (n = 2)	2 (100)	0	0	2 (100)	2 (100)	0	0	2 (100)	2 (100)	2 (100)	1 (50.0)	0	1 (50)
RR (mixed) (n = 8)	7 (87.5)	1 (12.5)	4 (50.0)	2 (25.0)	7 (87.5)	1 (12.5)	2 (25.0)	4 (50.0)	6 (75.0)	6 (75.0)	4 (50.0)	1 (12.5)	3 (37.5)
*p*-value	*0.074*	*1.000*	*0.549*	*0.004*	*0.009*	*1.000*	*0.657*	*0.017*	*1.000*	*0.202*	*1.000*	*1.000*	*0.051*

* T-bil↑: hyperbilirubinemia; ** H↑: hepatic enzymes elevated; *** P↑: pancreatic amylase elevated. MODs: multiple organ dysfunctions.

## Data Availability

All data generated or analyzed during this study are included in this article. The accession numbers of the obtained DNA sequences are mentioned in the Results and are available in the GenBank (https://www.ncbi.nlm.nih.gov/nuccore, accessed on 6 May 2023).
